# What are the prognostic factors affecting 30-day mortality in geriatric patients with respiratory failure in the Intensive Care Unit?

**DOI:** 10.12669/pjms.37.1.3189

**Published:** 2021

**Authors:** Mustafa Ozgur Cirik, Derya Yenibertiz

**Affiliations:** 1 Mustafa Ozgur Cirik, Department of Anesthesiology, University of Health Sciences, Ataturk Chest Diseases and Chest Surgery Training and Research Hospital, Ankara, Turkey; 2 Derya Yenibertiz, Department of Pulmonology, University of Health Sciences, Ataturk Chest Diseases and Chest Surgery Training and Research Hospital, Ankara, Turkey

**Keywords:** 30-day Mortality, Geriatric patient, Intensive Care Unit, Prognostic factors

## Abstract

**Objective:**

We aimed to investigate the prognostic factors related to 30 day mortality of elderly patients with respiratory failure in the intensive care unit (ICU).

**Methods:**

We performed a single centre, retrospective study and analyzed the main variables and outcomes of 238 geriatric patients admitted to an ICU with ARF between December 2017- January 2019 in Chest Disease Hospital, were included and classified as survivors and nonsurvivors. Main characteristics, laboratory datas, severity and nutrition scores were evaluated and logistic regression analysis were used.

**Results:**

The nonsurvivor group included 110 cases (40% female,) with a median age of 79, had higher scores in the followings; Acute Physiology Chronic Health Evaluation II score (APACHE-II) (p < 0.001), Charlson Comorbidity Index (CCI) (p < 0.001), Sequential Organ Failure Assessment score (p < 0.001). The inotropic support requirement was also higher in the nonsurvivor group (48,2%). As a comorbidity, malignancy and Type-I respiratory failure were higher in the nonsurvivor group (p=0.03, p < 0.001). The overall 30-day mortality was 46%. Blood urea nitrogen, procalsitonin, C-reactive protein and creatinine levels were higher in the nonsurvivor group (p < 0.001). However, albumin (p < 0.001), BMI (p=0.03) and longer hospital stay (p < 0.001) were higher in the survivor group. Inotropic support, APACHE-II score and CCI were independently related to increased mortality risk, whereas albumin was associated with decreased mortality risk.

**Conclusion:**

High APACHE II score, low CCI, low albumin levels and the requirement for inotropic support were found to be independently risk factors of 30-day mortality in the geriatric patients with respiratory failure in ICU.

## INTRODUCTION

With the increasing of longevity, the geriatric population has also been rising all over the world in the past few decades. The World Health Organization (WHO) declared that by 2050, approximately 16% of the world population, will be equal or more than 65 years old.^1^ These elderly population are representing majority of the patients admitted to ICU.^2^ The incidence of acute respiratory failure (ARF), the major indication of hospitalization in the ICU, increases exponentially with age.^3^-^5^ The relationship between prognosis and the age of the critically ill patients has been investigated in detail for the Iast three decades. Advanced aged patients had higher mortality rates than younger aged patients in prospective studies.^6^-^9^ It is known that geriatric patients are more likely to die in the ICU and age is independent risk factor for mortality.^10^ Diseases have atypical presentations in the geriatric population and there are lots of co-morbidities that increase the mortality rate for in this age group.

To determine the prognostic factors affecting mortality is crucial for the management of diagnosis and treatment of geriatric patients. Therefore, much more clinical studies and evaluations are needed to define the prognostic factors of geriatric patients with respiratory failure in the intensive care unit. We aimed to investigate the outcomes of geriatric patients treated for ARF in the ICU and to determine the prognostic factors related with 30-day mortality rate in this study.

## METHODS

We performed a single centre, retrospective study and analyzed the main variables and outcomes of 238 geriatric patients admitted to an ICU with ARF between December 2017- January 2019 in Chest Disease Hospital. The study was approved by the medical training committee of the hospital (approval number: 659) on 16th of January, 2020 and the informant consent was not obtained because of the retrospective nature of the study. Geriatric patients were defined as aged 65 years old and over. Patients, referred to another clinic or hospital during one month (n:27), died within 24 hours after admission (n:8) and had missing data (n:10) were excluded from the study. Age, gender, comorbidities, hospital stay in length in ICU, body mass index (BMI), duration of mechanical ventilation, re-admission to ICU, type of respiratory failure (Type-1 or Type-2), inotropic support, laboratory parameters at the time of diagnosis (biochemistry, hemogram, procalsitonin, C-reactive protein) were recorded from the patient files. Severity scores such APACHE-II score, the Sequential Organ Failure Assessment (SOFA) score, and Charlson Comorbidity lndex (CCI) and nutrition scores such as Nutrition Risk Screening (NRS) 2002; Nutrition Risk in Critically ill (Nutric Score) obtained within the first 24 hours of ICU admission. Patients were classified into two groups; survivors and non-survivors according to 30-day mortality. The thirty-day mortality was defined as mortality due to any cause during hospitalization in the ICU or after discharge from ICU to other medical ward or to home within 30 days.

### Statistical Analysis

The Statistical package program for the Social Sciences (SPSS) version 21.0 was used for the statistical analysis. The continuous variables were assessed by Kolmogorov-Smirnov test and histograms to find out if their distributions were normal or not. The normally distributed numerical parameters were analyzed by student’s t-test and others were analyzed by Mann-Whitney U test. The categorical variables were compared by Chi-squares or Fisher’s Exact test. A p < 0.05 was considered as statistically significant.

For the logistic regression analysis, univariate analysis was performed first, and accordingly multivariate logistic regression analysis with a backward stepwise approach were done. Hosmer-Lerneshow goodness of fit statistics were used to assess model fitness.

## RESULTS

Between December 2017 and January 2019, a total of 351 patients were admitted with the diagnosis of ARF in the one of the third level ICU of our hospital. A total of 238 geriatric patients found to be over 65 years old (male: 136, female: 102) analyzed in the study. Invasive mechanical ventilation was applied to 111 patients and non-invasive mechanical ventilation to 127 patients. Patients were classified into two groups survivors and non-survivors according to 30-day mortality. The main characteristics and laboratory data of the participants are shown in Table-I.

**Table-I:** Main characteristics and laboratory data of the patients.

	Survivors (n=128)	Non-survivors (n=110)	p-value
Age	77 (65-95)	79 (65-95)	0.05
Female sex. n, (%)	58 (45.3)	44 (40)	0.36
Malignancy. n, (%)	10 (7.8)	18 (16.8)	0.03
Charlson Comorbidity Index	6 (4-13)	7 (4-13)	<0.001
APACHE-2 score	20 (10-34)	26 (13-48)	<0.001
SOFA score	5 (4-8)	8 (4-17)	<0.001
BMI. kg/m^2^	26 (12-53.3)	24.15 (13.80-46.80)	0.03
NRS 2002 score	5 (2-6)	5 (4-6)	0.70
Inotropic support. n, (%)	12 (9.4)	53 (48.2)	<0.001
Length of stay in hospital (d)	16 (1-93)	11 (1-60)	<0.001
Length of stay in intensive care unit (d)	3 (1-50)	4 (1-34)	0.44
Re-admission to intensive care unit. n, (%)	19 (14.8)	9 (8.3)	0.11
Duration of mechanical ventilation (d)	3 (1-48)	3 (1-34)	0.556
Type-I respiratory failure, n, (%)	34 (26.6)	54 (49.1)	<0.001
Type 2 respiratory failure, n, (%)	94 (73.4)	56 (50.9)	<0.001
BUN, mg/dL	31 (10-137)	40,5 (10-125)	<0.001
Serum Creatinine, mg/dL	0.9 (0.3-6.5)	1.2 (0.4-5.2)	<0.001
GFR, ml/dk/1,73 m^2^	67 (5-116)	47.5 (9-105)	<0.001
Albumin, gr/dL	3.3 (1.6-4.7)	2.9 (1.6-4.2)	<0.001
CRP, gr/dL	3.9 (0.08-33.6)	8.5 (0.01-37.9)	<0.001
Procalcitonin, ng/mL	0.15 (0-25.3)	0.68 (0-97)	<0.001
Leukocytes, x 1000 cells/mm^3^	10.4 (3.5-97.5)	12 (0.15-110)	0.11
NRS 2002 score	5 (2-6)	5 (4-6)	0.70
Nutric score	5 (2-8)	7 (3-9)	<0.001

BMI, Body Mass Index, categorical variables were presented as n (%), skew distributed ones presented as median (min-max) APACHE-II, Acute Physiology and Chronic Health Evaluation-2; SOFA, The Sequential Organ Failure Assessment; BUN, Blood Urea Nitrogen; GFR, Glomerular Filtration Rate; CRP, C-Reactive Protein; variables were presented as median (min-max) NRS, Nutrition Risc Screening; Nutric, Nutrition Risk in Critically ill.

The survivor group included l28 patients (58 female, 45.3 %) with a median age of 77 (65-95 years) and non-survivor group consisted of 110 cases (44 female, 40%) with a median age of 79 (65-95 years). All co-morbidities of the patients were recorded and the number of accompanying malignancies was significantly higher in the non-survivors (p=0.03). Compared with the survivor group, non-survivor group had a higher CCI (p < 0.001), APACHE- II score (p < 0.001), SOFA score (p < 0.001). Almost half of the non-survivor group (48,2%) required inotropic support and it was significantly higher in non-survivor group (p < 0.001). Survivor group had a significantly higher BMl (p=0.03) and length of stay in hospital (p < 0.001). The 30-day mortality rate was 46% in ICU. While median BUN, creatinine, CRP and procalsitonin levels were significantly higher in the non-survivor group, median albumin level was significantly higher in the survivor group. NRS 2002 was similar in both groups but Nutric scor was significantly higher in the non survivor group.

The following factors such as BMI, presence of malignity, inotropic support, presence of Type-1 respiratory failure, APACHE-II score, CCI, Creatinine, CRP, albumin, were put into the multivariate logistic regression analysis to detect the possible independent parameters that affects 30-day hospital mortality. Variables eligible for inclusion in the multivariate analysis were tested for collinearity. Logistic regression analysis demonstrated that inotropic support, APACHE-II score, and CCI were independently related to increased mortality risk, whereas albumin was associated with decreased mortality risk. The results of logistic regression analysis are summarized in Table-II.

**Table-II:** Independent predictors of 30-day hospital mortality.

	*Unadjusted*		*Adjusted*	
Risk Factors	OR (95% CI)	p	OR (95% CI)	p
Inotropic support	8.98 (4.45-18.14)	< 0.001	5.29 (2.33-12.00)	< 0.001
APACHE-II	1.16 (1.10-1.22)	< 0.001	1.12 (1.06-1.19)	< 0.001
Charlson Comorbidity Index	1.60 (1.35-1.89)	< 0.001	1.25 (1.02-1.54)	0.032
Albumin	0.22 (0.12-0.38)	< 0.001	0.37 (0.19-0.73)	0.004

**OR:** odds ratio, 95% **CI:** 95% confidence interval; The p-value of the Hosmere-Lemeshow test was 0,255, the following factors were entered into the multivariate logistic regression analysis: BMI, presence of malignity, inotropic support, presence of Type-I respiratory failure, APACHE-II score, Charlson Comorbidity Index, Creatinine, CRP, albumin.

## DISCUSSION

Geriatric patients are frequent users of the intensive care units and a significant portion of these patients are over of the age 65 worldwide.^11^ We investigated the outcomes and the prognostic variables associated with mortality rate within 30 days of hospitalization in 238 geriatric patients (67, 6% of all patients) with ARF. We have identified many poor prognostic factors of those population, including lowness of BMI, albumin and GFR, accompany of malignancy as a comorbidity, presence of Type-I respiratory failure, receiving inotropic support and APACHE-II score, SOFA score, CCI, CRP and procalsitonine levels. Several studies have investigated the outcomes of critically ill elderly patients from a different perspective.^12^-^14^ In our study, we wanted to emphasize the prognostic factors in geriatric patients hospitalized with respiratory failure in the chest diseases hospital. We also consider that knowing the factors affecting the mortality of this specific patient group, which constitutes the majority of the intensive care patient population, affects the outcomes of the intensive care units. While 30-day mortality rate of the all patients of our ICU was 40%, it was 46% among geriatric patients. This rate was compatible with the literature, as in the study of Chih-Cheng Lai et al.^4^ Because our hospital is a chest disease hospital, all of our intensive care patient population consisted of those diagnosed with respiratory failure and as expected the mortality rate of this risky population is high. Nutritional assesment is a good predictor of the clinical outcome in the patients above the age of 65 admitted to the ICU. As in the study of Onal et al., low levels of BMI and albumin were found to be poor prognostic factors in determining the 30-day mortality of the geriatric patients in our study too.^15^ Nutrition Risk Screening-2002 (NRS-2002) and the Nutrition Risk in the Critically ill (NUTRIC) are major instruments for nutrition risk assessment in critically ill patients.^16^ We used either tool for evaluation of nutrition status.While NRS 2002 was similar in both groups, Nutric scor was significantly higher in the non survivor group. We think that Nutric score has better distinctive ability in determining mortality risk in intensive care patients due to including disease severity parameters.

The APACHE-II score is still effectively used in various patient groups to assess the severity and predict the prognosis of patients admitted to ICU. Several studies have reported the relationship between the mortality rate of critically ill patients and the APACHE-II score was found to be related with poor health status and a worse prognosis.^17^-^21^ The severity of illness during admission to ICU was assessed by using the APACHE II score system in our study and our findings were compatible with these studies. The CCI is first defined as an index of multiple comorbidities including 22 items to predict 1 year mortality in internal medicine patients.^22^ In our study, CCI was found as positively correlated with 30-day mortality (OR 1.25, p = 0.032). The SOFA score is used to assess organ dysfunction of six vital organs and higher scores are known to be associated with more severe disease and a higher mortality.^23^ In our study SOFA score was also correlated with 30 -day mortality (p < 0.001). Kuo-Chin Kao et al. and Katsutoshi Ando et al. reported that the CC1 and SOFA were significantly positively correlated with hospital mortality.^24^,^25^ We suggest that all these severity indices can be used in 30-day mortality estimation according to the clinician’s decision.

lnotropic support was found as independent risk factor related with adverse outcomes in geriatric patients admitted to ICU in Orsini J et al’s^26^ study and it was independently associated with 30-day mortality in our study too. The patients applied inotropic support should be followed closely in terms of early treatment and interventions.

PCT and CRP are well-known markers of bacterial infections, and can be used to predict increased mortality rate in critically ill patients. Nevertheless they were shown to be good diagnostic markers for patients with suspicion of sepsis and can prompt the severity of sepsis.^27^-^30^ The presence of sepsis is associated with extremely high mortality in ICU. As expected, the CRP and PCT levels were also detected to be higher in the non-survivor group but we didn’t evaluate sepsis rate in this study.

We think that this study is valuable as the factors affecting the 30-day mortality of geriatric patients in the respiratory ICU of the chest diseases hospital were evaluated and our results reinforce present evidence on predictive factors of mortality in geriatric patients treated in ICU.

### Limitations of the study

It was a retrospective study. Secondly, The sample size was relatively small and the study was conducted at a single center. We didn’t classified the geriatric patients into age groups and we didn’t determine the long-term survival of geriatric patients. We also didn’t evaluate the level of cognition functions of the geriatric patients because of the retrospective nature of the study.

## CONCLUSION

The 30-day mortality rate was 46% and risk factors associated with the higher mortality of specific population treated in ICU were higher APACHE scores, CCI, lower albumin and presence of inotropic support in this study. Our findings may help physicians predict 30-day mortality and also facilitate to manage the diagnose and treatment of geriatric patients with respiratory failure in ICU.
